# Resource Management Challenges in Rural Dermatological Care: A Mapping Review

**DOI:** 10.7759/cureus.77544

**Published:** 2025-01-16

**Authors:** Maria C Johnson, Priya Patel, Ashley Ayers, Kathleen M Spears

**Affiliations:** 1 Rural Medicine, University of Missouri Kansas City School of Medicine, St. Joseph, USA

**Keywords:** dermatology, primary care, rural dermatology, rural healthcare access, rural healthcare disparities

## Abstract

Skin cancer prevalence in the United States is rapidly on the rise, particularly in rural communities where individuals are subjected to heavy sunlight exposure through occupations such as agricultural work and construction. These geographic regions are often lacking in dermatologic specialty care, thus increasing the disease burden of skin conditions in primary care settings. Access barriers to specialized dermatology care are exacerbated by prolonged wait times to schedule an appointment, travel demands, and a relative paucity of dermatology providers in rural areas as compared to urban areas. In rural communities, the high burden of skin diseases, the logistical challenges, and the shortage of dermatologists lead to increased reliance on primary care physicians (PCPs) for dermatological care. This review aims to identify barriers to dermatology care in rural communities, understand challenges faced by PCPs related to dermatological management, and explore modalities that are currently being used to streamline clinical workflows for PCPs.

Dermatology training for PCPs consists primarily of pre-clinical exposure to the field, and it has been reported that there is a relative lack of opportunity for training to further enhance postgraduate dermatology knowledge. Recent studies demonstrate that novel educational and infrastructural support to primary care clinics has had substantial positive impacts on the ability of primary caregivers to provide accurate, cost-effective dermatologic care in addressing skin conditions, as well as increasing diagnostic confidence. Regular skin examinations, dermoscopy, and digital transformation of images are also shown to improve detection and diagnostic accuracy. Interventions like the use of smartphones, teledermatology, and dermoscopy show potential for improving care but must be thoroughly evaluated for effectiveness before widespread adoption in primary care settings.

## Introduction and background

One in five Americans will develop skin cancer by the age of 70 [1]. In the United States, more people develop skin cancer each year than all other cancers combined [1]. Recent studies also show an increased incidence of skin cancers in individuals who work in agriculture and construction, two occupations heavily represented in rural communities [2]. With the increasing prevalence of skin malignancies in rural areas and the relative lack of dermatology specialty care compared to urban regions, it is imperative to understand the workflow, screening techniques, and diagnostic workup of dermatological complaints performed in primary care clinics, which are often the first-line options for care for both benign and malignant skin conditions [1,2]. Rural healthcare systems are further challenged by low-resource environments, large geographic areas of impact, and diverse presentations of skin complaints, necessitating a hard look at the inefficiencies and areas of waste in the care access pathway(s) available to rural patients [3,4]. 

According to a report from the American Academy of Dermatology, 27% of the United States population was seen for a dermatological complaint in 2013 [5]. Among the subcategories of skin pathology, non-cancerous skin lesions were found to have the greatest prevalence of disease burden, at 7.81%, of all diagnosed cases that year [5]. Despite the low prevalence of melanoma, an invasive skin cancer of melanocyte origin, relative to other benign conditions, its incidence has been increasing over the last several decades [6]. Melanoma is a malignancy with high curative potential if treated at an early stage. Five-year survival rates were recently documented at over 99% in non-invasive disease but fell sharply to just 30% in melanoma cases with distant metastases [6]. 

It has also been found that individuals over the age of 65 years who were insured through Medicare accounted for nearly half of the prevalence of reported skin disease [6]. As the population ages and this demographic grows, it is expected that the skin disease burden will increase [7]. This represents a potentially unique challenge for healthcare providers of dermatology care in rural areas, as nearly 20% of the United States population lives in rural communities [8]. This evidence underscores the importance of timely, accurate dermatological disease management to reduce disease burden, especially in rural communities.

Individuals seeking care for skin disease in rural communities are disproportionately affected by barriers to healthcare access including, but not limited to, travel distance to acute and specialty care, relative poverty compared to larger metropolitan areas, low density of specialty care providers, and wait times for specialty referral [9-11]. There are 40 times more dermatology specialists in urban areas than in rural areas, which causes multiple barriers to care [9]. In major cities, patients wait an average of 32.3 days to see a dermatologist, a 46% increase since 2009. In rural areas, wait times can extend to several months due to the scarcity of practicing dermatologists, with fewer than 10% working in rural regions. Many rural patients face the added burden of traveling one to two hours for care [11]. Due to these factors, individuals from rural areas are 1.15 times more likely to present with later stages of melanoma than their urban counterparts [10]. This disparity underscores the need for improved access to dermatology services in underserved rural communities to prevent delays in treatment and the worsening of skin conditions. Primary care physicians (PCPs) subsequently find themselves managing the disease burden of skin conditions in these geographic areas amidst the management of a wide variety of competing disease processes.

According to the United States Preventive Services Task Force (USPSTF), regular screening for skin cancer is not a graded recommendation for clinical practice due to insufficient evidence of benefit [12]. As such, screening for skin disease is not a common tenet of preventative wellness screenings in the primary care setting. The mainstays of diagnostic evaluation for non-specific skin diseases or nevi, before excisional biopsy, include total body skin examination (TBSE), lesion-directed screening (LDS), and directed dermoscopy [13,14]. TBSE is a crucial diagnostic tool that enables early detection of serious skin conditions, leading to timely treatment and improved patient outcomes. It is typically conducted annually as part of a complete health checkup, either by a PCP or by a dermatologist. However, comprehensive skin screening is inconsistently performed in primary wellness exams, and disparities in specialty care access further contribute to variations in care. Among the most devastating preventable skin diseases routinely detected via TBSE is melanoma. Patients whose annual screening battery does not include a TBSE go without a critical organ system assessment, making them vulnerable to a highly treatable disease.

New techniques have emerged over the last 10 years for evaluating the influence of artificial intelligence (AI), digital manipulation of images, and spectroscopy for accurate diagnosis of skin diseases, specifically skin malignancies. Although evidence of the benefit of these emerging techniques is limited, utilization of techniques beyond individual physician assessment and before surgical excisional biopsy is vital to accurate and timely diagnosis of various skin ailments.

This research was previously presented as a poster at the 2023 Vijay Babu Rayudu Quality Improvement and Patient Safety Day on May 5, 2023.

## Review

This review was conducted using the keywords “skin cancer,” “rural medicine,” "primary care," and “dermatology.” A literature search was performed on PubMed, Springer Nature, and Google Scholar databases for articles published within the last 15 years. Randomized controlled trials, cohort analyses, case reports, and literature reviews that addressed the study question were included in the study.

The study team chose to perform a mapping review of the study topic to provide an overview of the extent, range, and nature of research on dermatology care in rural areas. Because this paper aims to summarize and qualitatively describe gaps and trends in the current climate of rural dermatology care and the interplay of primary care, statistical synthesis of data was not performed.

Barriers to access in rural communities

Geographic Barriers

Within the United States, the geographic location of patients relative to healthcare systems and clinics represents a large barrier to access [15,16]. It has been shown that patients in rural communities on average travel 2-3 times further for medical care than patients in urban communities [15]. In the state of Missouri alone, 55 of 114 counties do not have an active hospital, leaving almost half of the state without close access to acute care and further exacerbating the travel burden placed on patients [16]. These statistics reflect objective data and barriers patients face based on proximity to care, which may contribute to lower healthcare engagement and the increased rates of invasive disease observed in geographically isolated populations.

Economic Factors

The poverty crisis in many rural areas also creates barriers to healthcare access. There is a positive correlation between poverty and unmet medical needs in both rural and urban counties due to the lack of insurance coverage and lack of resources in these areas [17]. In 2019, the top 18 Missouri counties with the highest poverty rates were all defined as rural counties [16].

Federal initiatives through the Health Resources and Services Administration (HRSA) have spearheaded the provision of funds to health centers in rural communities to combat the relative lack of resources in rural health systems [2]. These interventions have been shown to reduce time delays in access to care, but have not been found to improve implementation of preventative screening measures [2].

Specialist Availability

A study performed in 2014 demonstrated that urban areas had a significantly increased density of gastroenterologists, radiation oncologists, and general surgeons compared to rural areas when understanding the availability of colorectal cancer care in the United States [9]. Physician density across all specialties and using national aggregate data was found to be 280 physicians per 100,000 people [9]. Stratification by area of practice found that rural areas had a density of 52 physicians per 100,000 people [9]. Specialist dermatology care was further found to be significantly underrepresented in rural communities, with 0.085 physicians per 100,000 as compared to 4.11 per 100,000 people in metropolitan areas [9]. 

Access barriers to specialized dermatology care are further compounded by referral wait times. There is an inverse relationship between the density of dermatology providers and wait times following referral [11]. The average maximum wait time for dermatology appointments in large metropolitan areas is up to 12.7 weeks, with wait times in rural areas reaching up to 13.6 weeks [18]. Increased appointment wait times have been linked to negative mental health effects for waiting patients, as well as influencing the likelihood that patients will actually attend their appointments [11]. In an analysis of the United Kingdom’s previous two-week rule for referral and management of new melanoma diagnoses, it was found that excision and treatment within two weeks correlated with improved patient outcomes and long-term survival [19]. It is clear from this data that minimizing patient wait times for dermatology care is essential for the optimization of disease management.

Physician education

Current Framework for Primary Care Dermatology Training

PCPs are often the first point of access for assessment of skin conditions, especially in rural areas. However, the current and chronic overburdening clinical load of most PCPs leaves them with minimal time for comprehensive preventative, chronic, and acute care. A recent study estimated that PCPs would require an average of 26 hours per day to provide comprehensive care amidst current guidelines and recommendations [20]. This problem is amplified in rural areas where there is a lower density of physicians, and, as follows, PCPs. Understanding the training and diagnostic confidence that PCPs have regarding the evaluation and management of skin conditions provides context into dermatological workup in rural areas where specialized dermatology care may not be immediately available. 

PCPs commonly complete a family medicine or internal medicine residency to earn board certification to practice. There is insufficient data regarding the dermatology curriculum in family medicine and internal medicine residency programs and therefore the average training a primary care physician receives in skin disease cannot be fully understood without further investigation. 

In most US medical schools skin disease is taught in the pre-clinical years of schooling, and a dermatology clerkship is not required for graduation. It is conceivable that such an approach may also have merit when applied to postgraduate continuing medical education opportunities. 

A needs assessment performed in Western India found that PCPs in both rural and urban clinical settings endorsed complications in dermatological management due to administrative factors, lack of opportunities for training, other collateral factors of skin diseases, and individual patient scenarios [21]. Subsequently, 70.5% of the physicians surveyed reported their dermatological training and knowledge as average and noted mitigation of these difficulties with dermatology referral to support the best patient outcome [21].

Reimagining Continuing Education for Dermatology

Independent, health system-wide initiatives for dermatology training have been implemented to provide better continuing education opportunities for PCPs in dermatology workup and management. A scoping review of sixteen dermoscopy training programs for primary care providers found that ten studies identified statistically significant changes in outcome variables ranging from diagnostic accuracy, and cost-effectiveness of dermatoscopy use, to confidence and attitudes [22]. This information underscores the opportunity afforded healthcare systems to enable inundated PCPs to excel in areas of skin management through educational and infrastructural support to the clinic.

Clinical decision making: screening, diagnostic tools, and referral workflow

Screening and Population Dynamics

The current USPSTF guidelines classify skin cancer screening as a Grade I recommendation, suggesting insufficient data to substantiate the benefit of screening, and as such, regular skin cancer screening practices and examination techniques are not part of the standard clinical workup [12]. Clinicians therefore often make use of techniques and protocols that they are most comfortable implementing and schemas with which they have the most experience; this mainly consists of the “ugly duckling” sign, the ABCDE rule of melanoma, and the Glasgow 7-point checklist [23]. Despite the absence of primary care clinical guidelines for skin checks, current practical recommendations for practitioners include behavioral counseling for children, adolescents, and young adults regarding avoidance of high-risk practices for skin cancer prevention [12]. 

Certain occupations, like agriculture and construction, have been found to benefit from similar behavioral counseling as a part of standard occupational safety. Agricultural workers are twice as likely to wear sun-protective clothing regularly than construction workers. However, only 20% of workers in both occupations reported regular use of sunscreen with SPF >45 [24]. Screening for skin conditions and associated behavioral counseling is imperative in the state of Missouri, where there are 92,000 farms comprising two-thirds of the state’s landmass and approximately one-third of the state's population [16,25]. 

Essential Elements of Skin Assessment

The mainstays of diagnostic evaluation for non-specific skin diseases or nevi, before excisional biopsy, include TBSE, LDS, and dermoscopy [13,14]. Regular TBSE, when performed at the primary care level, has been shown to reliably identify uncomplicated melanoma lesions [26]. Melanoma continues to be one of the most common and treatable skin malignancies when caught at an early stage [27], underscoring the importance of regular screening for skin cancer detection. A study performed in Sweden identified that patients were at greater risk for omitted TBSE if their presenting concern was not for a skin check which was hypothesized to be due to lack of time during the primary care visit to do a thorough evaluation and lack of screening for skin cancer risk [13]. Lesion-directed screening, rather than TBSE, is often found to be more frequently utilized due to the focused nature of the exam and the brevity in the context of limited time to address all patient concerns in primary care. 

Optimizing Dermoscopy for Improved Outcomes

Proper usage of dermoscopy has been shown to effectively approximate margins of melanoma before removal, as well as further classify nevi based on structural elements [14]. Diagnostic accuracy is optimized when physicians receive direct training with the dermatoscope. A review of dermoscopy training programs across Europe found post-graduate training of PCPs in dermoscopy to be feasible [22]. Summative evidence was insufficient to understand whether these training programs led to the regular adoption of dermoscopy in their daily practice [22]. Further, longitudinal outcomes were not assessed and it is unclear if these physicians would continue to refer their patients to dermatology.

Further analysis of these techniques can be performed using digital transformation of images taken during the initial examination. Sequential digital dermoscopy and computer-aided multispectral digital analysis provide mechanisms to analyze images and search nevi for concerning characteristics [28]. Computer-assisted diagnosis using artificial intelligence shows promise for diagnostic accuracy when combined with dermoscopy and physician evaluation. Computer-assisted diagnosis techniques and software vary greatly across the world but overall have been shown to detect cutaneous melanoma with a sensitivity of 90.1% and specificity of 74.3% when performed on dermoscopic images [3]. Sequential digital dermoscopy utilizes longitudinal monitoring of dermoscopic images to understand significant changes from baseline in patients with multiple cutaneous nevi [3]. Limitations of computer-assisted techniques involve low specificity of diagnosis, while sequential dermoscopy is limited by the number of data points needed to conclusively predict the nature of a lesion.

Referral Workflow and General Treatment Choices

The referral system is crucial to the coordination of care between medical specialties and the advocacy of various patient needs. Many PCPs rely on the referral system for the management of skin conditions amidst a lack of time for workups, gaps in knowledge, and administrative difficulties [21]. 

Despite the benefit of specialty care referral, barriers exist in the completion of a referral appointment from primary care. A large single-site study performed at Duke University found that 38.9% of referrals from primary care clinics were not associated with an appointment date [29]. Of the appointments that were scheduled, 56.9% were completed and therefore closed the referral loop [29]. Prolonged wait times are likely to exacerbate this issue when applied to rural health systems. A report produced by the Greater Access for Patients Partnership (GAPP) found that longer wait times for dermatology assessment were negatively correlated with subsequent appointment compliance when the patient was referred from another specialty [11].

Issues related to referral wait times may involve the volume of referrals from primary care to dermatology, and subsequent scheduling may be complicated by the presence of cases that may not need specialist attention. Triage efforts to mitigate redundant or unnecessary referrals may provide a feasible alternative to significant dermatology wait times. A teledermatology triage system was implemented in Sao Paulo in 2018 that utilized 13 dermatologists who assessed 55,000 lesions during the study period via images and phone calls with consenting patients [4]. The study found that 53% of lesions were referred back to primary care, 43% were referred to in-office dermatology, and 4% were referred directly for biopsy [4]. 64% of patients received medication following teledermatology assessment, the majority of which involved prescription of emollients [4].

Clinical Resource Management Strategies

Rapid access clinics in dermatology have also been shown to mitigate challenges related to increased referral volume and prolonged wait times. The University of Pennsylvania implemented urgent care and intermediate care to provide quicker access to dermatology care that was stratified by the severity of the condition [30]. Wait times were found to be 3 days and 31 days for urgent care and intermediate care, respectively [30]. The referral loop was closed for 36% of referrals across urgent, intermediate, and routine care referrals for dermatology, with the highest rate of appointment completion in urgent care with 64.9% completion [30].

Discussion

Summary of Findings

The purpose of this review was to identify barriers to dermatology care in rural communities and to understand the role of the primary care physician in the management of skin disease. Rural areas have increased healthcare resource needs due to many factors that create access barriers including relative poverty, paucity of specialty care providers, increased disease burden on primary care providers, lack of medical facilities, greater travel times to seek care, and vulnerable populations living in these areas [9,11,16,17]. Rural communities are disproportionately affected by barriers to dermatology care specifically as there are 0.085 dermatology providers available per every 100,000 people in rural U.S. areas, amplifying existing access barriers.

PCPs play a pivotal role in the evaluation and management of skin disease, especially in rural areas. Due to the high disease burden that rural PCPs must balance, there exists a need to efficiently streamline their clinical decision-making process to facilitate the management of skin disease. Referrals to specialty care circumvent this issue well. Nevertheless, referral wait times create a barrier to dermatology access and are found to be up to 13.6 weeks in rural communities [18]. Long wait times are inversely correlated with appointment completion in dermatology. Studies have shown that approximately 36-39% of all dermatology referral appointments are completed [29,30] which necessitates a focused look at the referral process and workup before referral to achieve better access to first-line treatment for patients with skin disease. 

**Figure 1:**
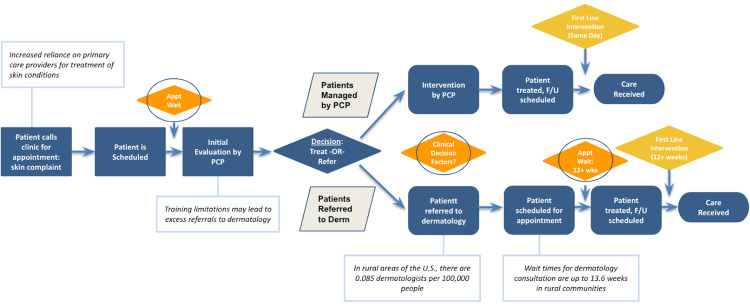
Process flow map illustrating the clinical workflow for the care for skin conditions from primary care to dermatology This figure was created by the authors using digital software F/U: follow-up; PCP: primary care physician

Informed by the literature review performed for this paper as well as anecdotal experiences of the authors’ work in primary care, a process flow map was created to visualize the workflow from the initial presentation of patients with skin complaints to dermatology and/or primary care management (Figure 1). Clinical decision-making factors including screening guidelines, availability of clinical tools, and physician confidence were identified along with appointment wait times to be areas of improvement to provide more readily accessible dermatological care in rural communities.

Mainstays of dermatology workup in the primary care setting include dermoscopy, TBSE, pharmacotherapy, and referral to dermatology or teledermatology in cases that are refractory to initial treatment. There is insufficient data to understand the level of training that PCPs receive in these diagnostic tools and clinical diagnosis of skin disease beyond the medical school pre-clinical curriculum. Training models have been implemented in Europe that show a greater understanding of these diagnostic practices in PCPs, however long-term outcome data for behavior change and implementation in practice is not well understood.

In-person evaluation of skin lesions by dermatologists has significantly greater diagnostic accuracy than primary care or teledermatology evaluation [4]. Smartphone applications, teledermatology, AI supplemental technology, and training in dermoscopy at the primary care level may shorten wait times to further dermatology management and help distinguish the severity of lesions. A discussion regarding the utilization of interventional tools must include the context of the training provided to PCPs to understand the optimal efficacy of these modalities. Any interventions to improve dermatological management should aim to be efficient, and feasible, and require minimal additional training.

Limitations

Cutaneous melanoma is used as a proxy throughout this paper to understand disease prevalence and patient outcome data. The study team recognizes that disease prevalence varies for different types of skin malignancies and benign conditions. Melanoma is a widely studied disease and there is significant data regarding patient outcomes. Further, the disease process and potential for progression contextualize the importance of timely dermatological intervention. 

The presentation of data regarding physician confidence and diagnostic accuracy is limited by the paucity of studies on the topic and may not be generalizable to all PCPs given the variability in curriculum for individual post-graduate residency training programs and medical school curricula.

Areas of Future Research

Future studies should work to further quantify the disease burden, morbidity, mortality, and patient costs of dermatologic conditions in rural healthcare settings as they compare to their urban counterparts. Further, quality improvement initiatives would be beneficial to assess the needs of rural PCPs in the United States to propose interventions needed to facilitate efficient skin disease diagnosis and management. Structured interviews with PCPs and dermatologists in rural areas would be an effective way to characterize the needs of these practice settings. The focus of this review was to examine the relationship of primary care to dermatology with an emphasis on primary care workup due to the limited specialty availability in rural settings. It would be important to explore the perspective of dermatology in future research to understand the necessity of referrals from all specialties for skin conditions to better streamline the clinical referral workflow while optimizing the time of dermatologists.

## Conclusions

A high rural skin disease burden coupled with a paucity of dermatology specialists has led to increased reliance on PCPs for the evaluation and management of skin conditions. Increased disease burden from all causes on PCPs, lack of standardized training in primary care, and lack of specialist availability lead to increased referrals and contribute to longer patient wait times for dermatology management. Interventions via smartphone utilization, dermoscopy training, implementation of teledermatology, and training in TBSE have shown promise in improving physician confidence and streamlining skin disease workflow in the rural primary care setting. These interventions and other emerging techniques must be critically evaluated to better understand clinical benefits, feasibility, and cost-effectiveness before their adoption at the primary care level.
